# Cracking the code of cellular protein–protein interactions: Alphafold and whole‐cell crosslinking to the rescue

**DOI:** 10.15252/msb.202311587

**Published:** 2023-03-10

**Authors:** Toni Träger, Panagiotis L Kastritis

**Affiliations:** ^1^ Interdisciplinary Research Center HALOmem, Charles Tanford Protein Center Martin Luther University Halle‐Wittenberg Halle/Saale Germany; ^2^ Institute of Biochemistry and Biotechnology Martin Luther University Halle‐Wittenberg Halle/Saale Germany; ^3^ Biozentrum Martin Luther University Halle‐Wittenberg Halle/Saale Germany; ^4^ Institute of Chemical Biology National Hellenic Research Foundation Athens Greece

**Keywords:** Microbiology, Virology & Host Pathogen Interaction, Proteomics, Structural Biology

## Abstract

Integration of experimental and computational methods is crucial to better understanding protein–protein interactions (PPIs), ideally in their cellular context. In their recent work, Rappsilber and colleagues (O'Reilly *et al*, 2023) identified bacterial PPIs using an array of approaches. They combined whole‐cell crosslinking, co‐fractionation mass spectrometry, and open‐source data mining with artificial intelligence (AI)‐based structure prediction of PPIs in the well‐studied organism *Bacillus subtilis*. This innovative approach reveals architectural knowledge for in‐cell PPIs that are often lost upon cell lysis, making it applicable to genetically intractable organisms such as pathogenic bacteria.

## Graphical abstract

Integrative approaches are crucial for identifying protein‐protein interactions (PPIs). In their recent work, Rappsilber and colleagues (O'Reilly *et al*, 2023) identify bacterial PPIs in their cellular context, by combining whole‐cell crosslinking, co‐fractionation mass spectrometry and AI‐based PPI structure prediction.

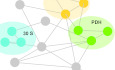

Recent developments in AI have made it possible to accurately model protein structures, resulting in a transformational breakthrough in molecular biology (Jumper *et al*, 2021). The applications have been numerous and include an open‐access database of 200 million Alphafold models (https://alphafold.ebi.ac.uk/), new avenues toward functional protein design via protein hallucination (Anishchenko *et al*, 2021), and the structural analysis of enigmatic cellular assemblies, such as the nuclear pore complex (Mosalaganti *et al*, 2022) and metabolons (Bourque *et al*, 2022). AlphaFold‐Multimer has taken the protein interaction field by storm, by predicting structures of PPIs at higher accuracy than previous methods (preprint: Evans *et al*, 2022), provided that the interaction interface is not mediated by hypervariable sequence regions. The fact that most previously experimentally determined PPIs lack structural information hinders functional studies and therefore developing methods that could speed up their determination is important. However, while remarkable progress has been made toward 3D modeling of PPIs (Bouatta & AlQuraishi, 2023), large‐scale structural prediction of biomolecular interactions remains computationally challenging. For a proteome consisting of *N* proteins, potential binary PPI predictions will scale with the power of two (*N* ^2^/2).

The authors leveraged these limitations into strengths. They generated a comprehensive list of high‐confidence PPIs from the open‐access database SubtiWiki (http://subtiwiki.uni‐goettingen.de/). After removing structurally known or homologous interactions, they ended up with 1,218 previously known PPIs with no high‐quality structural information. Many of these come from PPI mapping techniques such as affinity pulldowns so it is unknown which ones involve direct interactions. The authors also performed their own PPI mapping and identified 909 PPIs by whole‐c*ell* crosslinking mass spectrometry and co‐fractionation analysis (Fig 1A). Surprisingly, there was limited overlap across datasets, resulting in 2032 PPIs in total. The limited overlap indicates the complementarity of the approaches and possibly suggests that a vast number of PPIs remain to be discovered. It is of note that the Rappsilber laboratory, in collaboration with EMBL Heidelberg, previously performed crosslinking in native cell extracts of a eukaryote (Kastritis *et al*, 2017), as well as whole‐cell crosslinking in *Mycoplasma pneumoniae* (O'Reilly *et al*, 2020), proving the transferability of crosslinking methods to various organisms in the tree of life.

**Figure 1:**
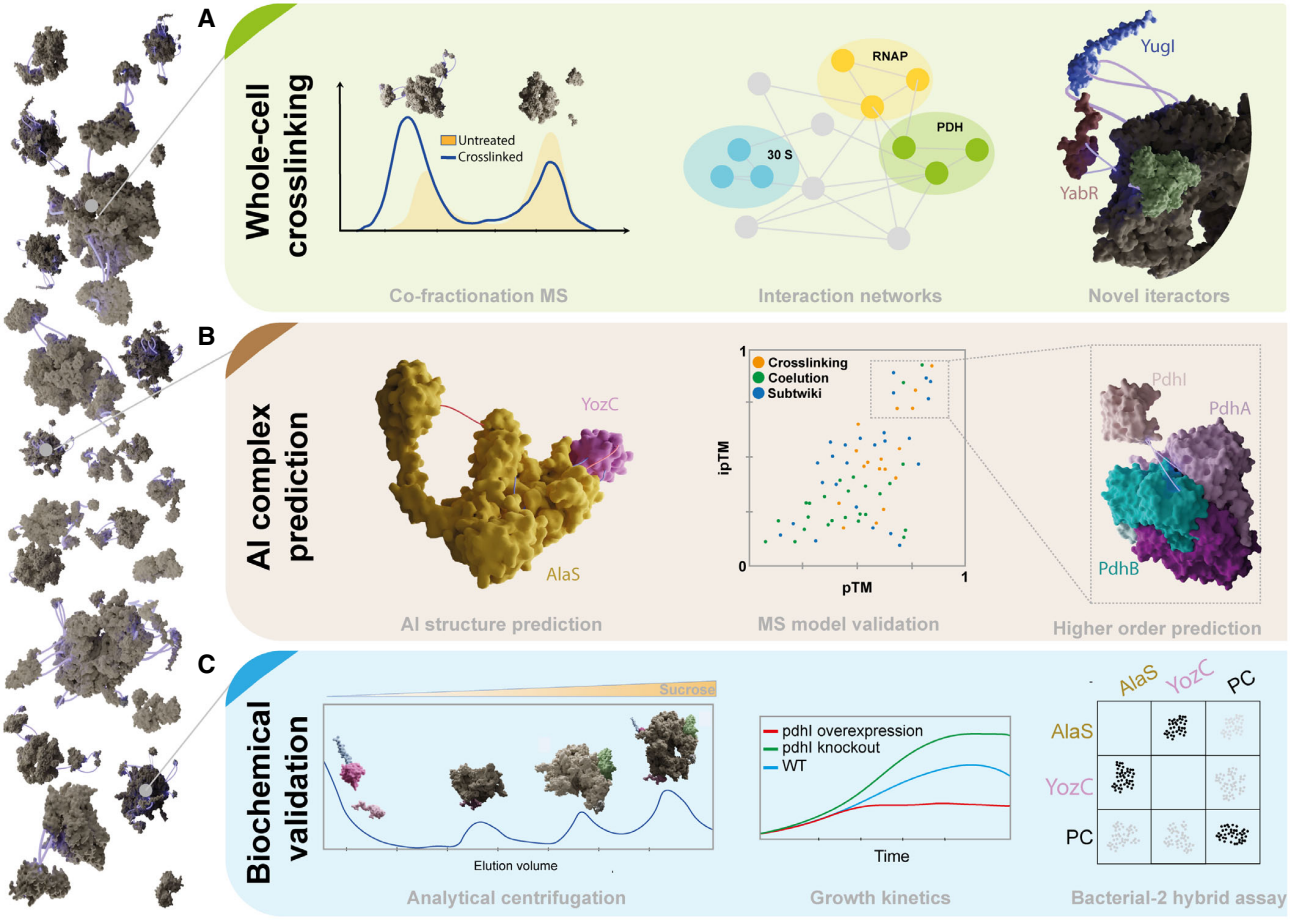
The Integrative workflow to interrogate cellular protein–protein interactions used by O'Reilly *et al* (2023) (A) Protein complexes were crosslinked and evidence was provided that in‐cell interactions are retained and can be annotated using co‐fractionation mass spectrometry, network biology, and crosslinking mapping. (B) These interactions were modeled and predicted using crosslink‐scored Alphafold models, providing functional hypotheses for the direct interaction of unknown proteins in *Bacillus subtilis*. (C) Biochemical validation of novel complexes involved measurements of analytical ultracentrifugation, growth strain kinetics, and bacterial 2‐hybrid assays.

As a next step, the authors predicted the structures of these 2032 PPIs using AlphaFold‐Multimer to reveal direct interactions and suggest functions (Fig 1B). An interesting relation emerged: the number of satisfied crosslinks positively correlated with measures for protein model quality. This finding is of significance, not only for the models' accuracy but also for structural biology in general, as it shows that structural data deposited in the open‐access Protein Data Bank (PDB, https://www.rcsb.org/) which Alphafold used for training (i.e., ground truth) may approximate in‐cellular conformers as crosslinks are not violated.

The authors then selected interactions from the pool of highest‐quality predictions to validate Alphafold models via functional experiments (Fig 1C). The predicted direct interaction of two proteins, YozC and alanine‐tRNA synthetase (AlaS), was confirmed by a bacterial‐2‐hybrid assay in *Escherichia coli*. The predicted structures also suggested a potential protein regulator (Pdhl) of the activity of the pyruvate dehydrogenase subunit E1p. To check its function, O'Reilly *et al* generated two strains, one that overexpressed PdhI and one where PdhI was knocked out. They observed that the strains did not show growth defects compared to the wild type when grown on glucose as main carbon source. However, cells overexpressing PdhI showed growth defects when grown on pyruvate as the sole carbon source, indicating that PdhI acts as an inhibitor of pyruvate dehydrogenase. Point mutations suggested by the Alphafold models were able to rescue this phenotype, highlighting the usefulness of using these predicted structures to understand function.

Overall, the findings of O'Reilly *et al* have significant implications for systems biology as identifying PPIs in their native context provides valuable insights into the functions of these interactions. The combination of data mining, crosslinking mass spectrometry, and co‐fractionation mass spectrometry, followed by computational modeling using AlphaFold‐Multimer and functional validation, offers a powerful approach for characterizing PPIs, particularly for genetically intractable organisms. The next big challenge in structural systems biology lies in improving the depths of such analyses, predicting and validating stoichiometries on large scale, and adding strong visualization, such as data from cryo‐electron microscopy, super‐resolution microscopy, and other multi‐scale approaches to further systems‐based understanding of biomolecular interactions, a key to understand life itself.
